# Impact of Sarcopenia and Its Severity on Postoperative Functional Decline in Patients Undergoing Cardiac Surgery

**DOI:** 10.31662/jmaj.2026-0041

**Published:** 2026-05-08

**Authors:** Daisuke Ishiyama, Yoshiaki Kubota, Masahiro Koen, Aya Moroi, Momoe Ono, Hayato Watanabe, Kaito Sakai, Yukihiro Watanabe, Hitomi Ueda, Kuniya Asai, Yosuke Ishii, Yoichiro Aoyagi

**Affiliations:** 1 Department of Rehabilitation Medicine, Nippon Medical School Hospital, Tokyo, Japan; 2 Department of Cardiovascular Medicine, Nippon Medical School, Tokyo, Japan; 3 Department of Cardiovascular Surgery, Nippon Medical School, Tokyo, Japan

**Keywords:** activities of daily living, cardiac surgery, rehabilitation, sarcopenia

## Abstract

**Introduction:**

In this study, we aimed to evaluate the impact of sarcopenia and its severity on postoperative functional decline in patients undergoing cardiac surgery.

**Methods:**

This retrospective cohort study included 161 patients (median age, 71 years; 75.8% male) who underwent elective cardiac surgery at a tertiary care hospital between April 2021 and November 2024. Functional decline was assessed by subtracting the preoperative Barthel Index (BI) score from the score at discharge. Sarcopenia severity was assessed based on the Asian Working Group for Sarcopenia 2019 criteria. Multiple linear regression models adjusted for relevant clinical and demographic factors were used to examine the associations among sarcopenia, severe sarcopenia, and functional decline.

**Results:**

The mean (standard deviation) change in BI score was −1.4 (5.7). The overall prevalence of sarcopenia was 21.1%, comprising 7.5% (non-severe) sarcopenia and 13.7% severe sarcopenia cases. In the multivariate analysis, the presence of sarcopenia was significantly associated with postoperative functional decline (β = −0.185, p = 0.001), whereas severe sarcopenia exhibited a stronger association with postoperative functional decline (β = −0.268, p < 0.001).

**Conclusions:**

These findings indicate that patients with severe sarcopenia require careful attention because of their higher risk of postoperative functional decline.

## Introduction

Cardiac surgery imposes significant physiological stress on patients. Perioperative management, including rehabilitation, plays a critical role in preserving physical function and in optimizing recovery. Postoperative complications, physical decline, and prolonged hospital stays are common after cardiac surgery, and can greatly impact healthcare resource utilization and post-discharge quality of life ^(1), (2), (3)^. In particular, older adults and patients with comorbidities often experience delayed progress in postoperative rehabilitation and reduced activities of daily living (ADLs) at discharge ^(4), (5), (6)^. These challenges highlight the necessity for structured perioperative care, including early mobilization and individualized rehabilitation, as essential components of comprehensive strategies to support recovery and maintain functional independence.

Postoperative functional decline is a major concern during cardiac surgery. This refers to a state in which ADLs decline compared to before surgery, which cannot be directly attributed to the underlying disease ^(7)^. Postoperative functional decline has been recognized as a major predictor of poor outcomes after cardiac surgery ^(8), (9)^, emphasizing the importance of preoperative risk assessment. Physical function-focused assessments are particularly useful for the early identification of patients at-risk for postoperative functional decline ^(10), (11), (12)^. Timely and targeted interventions based on such assessments may help prevent postoperative functional decline, promote patient independence, and improve postoperative outcomes.

Sarcopenia may have a significant effect on postoperative functional decline. Defined by reduced skeletal muscle mass and impaired muscle function, sarcopenia has been associated with increased mortality among older adults and individuals with wasting conditions ^(13), (14), (15), (16)^. Sarcopenia contributes to postoperative disability, complications, prolonged hospitalization, and high mortality rates ^(17), (18), (19), (20), (21), (22)^. Therefore, assessment and management of sarcopenia are critical components of perioperative clinical care.

In recent years, consensus-based diagnostic criteria for sarcopenia have been established along with a classification of its severity ^(23)^. However, the impact of sarcopenia and its severity on postoperative functional decline in patients undergoing cardiac surgery remains unclear. We hypothesize that preoperative sarcopenia and its progression to severe sarcopenia adversely affect postoperative functional decline. Therefore, in this study, we aimed to evaluate the impact of preoperative sarcopenia and its severity on postoperative functional decline in patients undergoing cardiac surgery.

## Materials and Methods

### Study design and participants

In this retrospective observational study, we included patients who underwent elective cardiac surgery for ischemic heart disease or valvular disease at the Nippon Medical School Hospital (Bunkyo-ku, Tokyo, Japan) between April 2021 and November 2024. Patients with cardiovascular implantable or wearable electronic devices (owing to contraindications for bioelectrical impedance), postoperative stroke complications, in-hospital death, and missing data were excluded. The study was conducted in accordance with the principles of the Declaration of Helsinki. The study protocol was reviewed and approved by the Institutional Committee on Human Research at the study facility (approval no. B-2020-325). Informed consent for this study was obtained using the opt-out method of poster announcements at Nippon Medical School Hospital.

### Study outcomes

Functional outcomes were assessed using the Barthel Index (BI) ^(24)^. The BI includes 10 items related to ADLs, with the maximum score for each item indicating full independence. Each item has a minimum and maximum score: feeding (0-10), transfers (0-15), grooming (0-5), toilet use (0-10), bathing (0-5), mobility (0-15), stairs (0-10), dressing (0-10), bowels (0-10), and bladder (0-10). Total BI scores range from 0 to 100 points. We calculated the change in the BI score by subtracting the preoperative BI score from the score at discharge. In addition, we assessed hospitalization-associated disability (HAD), defined as a decrease of at least 5 points in the BI score at discharge compared with the preoperative BI score ^(12), (25)^.

### Sarcopenia definition

Sarcopenia and its severity were defined using the Asian Working Group for Sarcopenia (AWGS) 2019 diagnostic criteria ^(23)^. We assessed appendicular skeletal muscle mass (ASM), muscle strength, and physical performance to diagnose sarcopenia a few days before surgery. ASM was assessed using Skeletal Muscle Mass Index (SMI) with a commercially available multifrequency bioelectrical impedance data acquisition system (Inbody S10; Inbody Japan Inc., Tokyo, Japan). Measurements were performed with patients in the supine position after a 15-min rest and a 2-h fast. The SMI was calculated as the muscle mass of the four extremities (estimated by lean mass of each) divided by height squared (kg/m^2^). Low ASM was defined as <7.0 kg/m^2^ for male and as <5.7 kg/m^2^ for female individuals. Muscle strength was assessed by measuring handgrip strength. Handgrip strength was measured twice on the patient’s dominant side while in the standing position using a Smedley hand dynamometer (TKK 5401; Takei Scientific Instruments Co., Ltd., Tokyo, Japan). Low muscle strength was defined as handgrip strength <28 kg for male and <18 kg for female individuals. Physical performance was assessed based on gait speed, which was measured using a 5-m usual gait speed. Patients walked 11 m at their usual pace. The time required to walk 5 m (between the 3-m and 8-m points) was measured using a stopwatch. The gait speed was calculated as 5 m divided by the time required (m/s). The best value of two measurements was used as the representative value of gait speed. Low physical performance was defined as <1.0 m/s gait speed. Trained physical therapists assessed muscle strength and physical performance. We defined sarcopenia as low ASM plus low muscle strength or low physical performance, and severe sarcopenia as low ASM plus low muscle strength and low physical performance.

### Demographic and clinical characteristics

The demographic and clinical characteristics included age, sex, underlying disease (ischemic heart disease or valvular disease), body mass index (BMI), comorbidities, use of walking aids, preoperative cardiac and respiratory functions, preoperative blood biochemical data, surgical details, and postoperative data. The comorbidities included hypertension, diabetes mellitus, dyslipidemia, stroke, and heart failure. Preoperative cardiac and respiratory functions included New York Heart Association (NYHA) classification, left ventricular ejection fraction, vital capacity (VC), forced expiratory volume in 1 s (FEV1), and FEV1/VC ratio. Preoperative blood biochemical data included N-terminal pro-brain natriuretic peptide (NT-proBNP) level, estimated glomerular filtration rate (eGFR), and serum albumin level. In addition, we calculated the Geriatric Nutritional Risk Index (GNRI) using serum albumin levels and BMI ^(26)^. Surgical details included surgical procedures, such as isolated coronary artery bypass grafting (CABG), isolated heart valve surgery, and combined procedures, and intraoperative factors, including operative time, intraoperative blood loss, and fluid balance. The postoperative data included complications, duration of mechanical ventilation, length of intensive care unit (ICU) stay, and length of hospitalization. Postoperative complications, defined according to the criteria of the Japanese Society for Cardiovascular Surgery, comprised renal failure requiring renal replacement therapy, reoperation for bleeding, cardiac arrest, atrial fibrillation, prolonged mechanical ventilation, reintubation, tamponade, septicemia, and gastrointestinal complications. A composite complication was defined as the occurrence of any of these complications.

### Statistical analysis

First, differences between the sarcopenia and non-sarcopenia groups were evaluated using the chi-square test for categorical variables and the unpaired t-test for normally distributed continuous variables or the Mann-Whitney U test for non-normally distributed continuous variables. In addition, differences among three groups (non-sarcopenia, sarcopenia, and severe sarcopenia) were evaluated using the chi-square test for categorical variables and the one-way analysis of variance (ANOVA) or the Kruskal-Wallis test for continuous variables, as appropriate. When significant differences were detected, post-hoc comparisons were performed using the Bonferroni test. Second, we conducted a two-way ANOVA to examine the relationship between the presence of sarcopenia and the time from the preoperative period to discharge. Third, the incidence of HAD in patients with and without sarcopenia was compared using the chi-square test. In addition, patient characteristics were compared between individuals with and without HAD using the chi-square test for categorical variables and unpaired t-test and Mann-Whitney U test for continuous variables, as appropriate. Finally, univariate and multivariate linear regression models were used to examine the association between changes in BI score and sarcopenia. In addition, we examined the association between changes in BI score and severe sarcopenia. The covariates included in the multivariate models were selected a priori based on clinical relevance and prior literature and comprised age, sex, preoperative BI, NYHA class, VC, operative time, and composite postoperative complications ^(9)^. Statistical significance was set at p < 0.05. All analyses were conducted using SPSS software (version 21.0; IBM Corp., Armonk, NY, USA).

## Results

**Figure 1.**
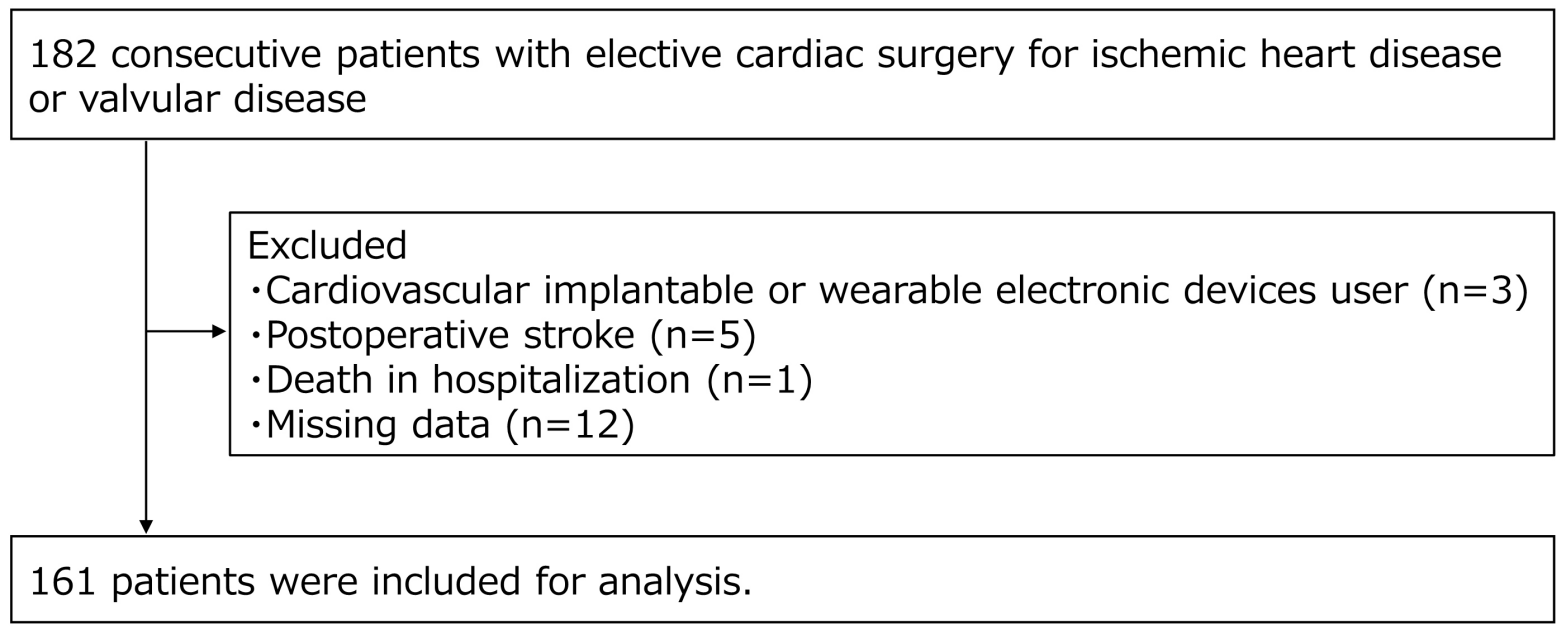
Flow diagram illustrating the patient selection process.

Of the 182 eligible patients, 21 were excluded because of cardiovascular implantable or wearable electronic device use (n = 3), postoperative stroke (n = 5), in-hospital death (n = 1), and missing data (n = 12: BI score, n = 3; SMI, n = 1; handgrip strength, n = 4; respiratory function, n = 1; and serum albumin, n = 3). Therefore, 161 patients were included in the analysis (Figure 1). In addition, none of the patients excluded from the analysis due to stroke (n = 5) or in-hospital death had sarcopenia at baseline.

The characteristics of all patients and comparisons of the variables according to sarcopenia severity are shown in Table 1, and those according to sarcopenia severity are shown in Online Resource 1. The median (interquartile range [IQR]) age of the patients was 71 (64-75) years, and 75.8% were male. Cardiac procedures included isolated CABG (31.7%), isolated valve surgery (42.2%), and combined surgery (26.1%). The median preoperative BI score was 100 (IQR, 100-100), with a median mobility subitem score of 15 (IQR, 15-15), indicating that all patients were independently mobile. Walking aids were used by 3.1% patients (n = 5), all of whom used a cane. The mean (standard deviation) change in BI score was −1.4 (5.7). The overall prevalence of sarcopenia was 21.1%, comprising 7.5% (non-severe) sarcopenia and 13.7% severe sarcopenia cases. Significant differences were observed between the sarcopenia and non-sarcopenia groups in age, BMI, heart failure, NYHA classification, NT-proBNP, eGFR, GNRI, SMI, handgrip strength, gait speed, preoperative BI score, use of walking aid, VC, FEV1, complications (cardiac arrest and prolonged mechanical ventilation), and length of ICU stay (p < 0.05). In addition, significant differences were observed according to sarcopenia severity in age, BMI, heart failure, NYHA classification, NT-proBNP, GNRI, SMI, handgrip strength, gait speed, preoperative BI score, use of walking aids, VC, FEV1, complications (cardiac arrest, prolonged mechanical ventilation, and tamponade), and length of ICU stay (p < 0.05).

**Table 1.** Patient Characteristics.

	Overall (n = 161)	Non-sarcopenia (n = 127)	Sarcopenia (n = 34)	p
Age (years), median (IQR)	71 (64-75)	70 (61-74)	74 (70-78)	0.001
Sex, male, n (%)	122 (75.8%)	99 (79.2%)	21 (61.8%)	0.174
Underlying disease, n (%)				0.190
Ischemic heart disease	53 (32.9%)	45 (35.4%)	8 (23.5%)	
Valvular disease	108 (67.1%)	82 (64.6%)	26 (76.5%)	
Cardiac procedures, n (%)				0.778
Isolated CABG	51 (31.7%)	43 (33.9%)	8 (23.5%)	
Isolated heart valve surgery	68 (42.2%)	51 (40.2%)	17 (50.0%)	
Combined	42 (26.1%)	33 (26.0%)	9 (26.5%)	
BMI (kg/m^2^), median (IQR)	23.7 (21.6-25.6)	24.3 (22.1-26.5)	21.4 (18.5-22.9)	<0.001
Comorbidity, n (%)				
Hypertension	91 (56.5%)	73 (57.5%)	18 (52.9%)	0.782
Dyslipidemia	78 (48.4%)	64 (50.4%)	14 (41.2%)	0.854
Diabetes	45 (28.0%)	36 (28.3%)	9 (26.5%)	0.096
Stroke	18 (11.2%)	11 (8.7%)	7 (20.6%)	0.080
Heart failure	37 (23.0%)	21 (16.5%)	16 (47.1%)	<0.001
NYHA classification, n (%)				0.011
I	40 (24.8%)	37 (29.1%)	3 (8.8%)	
II	118 (73.3%)	89 (70.1%)	29 (85.3%)	
III	3 (1.9%)	1 (0.8%)	2 (5.9%)	
LVEF (%), median (IQR)	64 (53-69)	64 (54-69)	65 (53-76)	0.340
NT-proBNP (pg/mL), median (IQR)	486 (159-1260)	447 (122-1136)	814 (301-2148)	0.007
eGFR (mL/min/1.73 m^2^), median (IQR)	60 (43-70)	62 (48-70)	53 (38-67)	0.034
Serum albumin (g/dL), median (IQR)	4.3 (4.0-4.5)	4.3 (4.0-4.6)	4.3 (3.9-4.5)	0.238
GNRI, mean (SD)	109.4 (9.9)	110.0 (9.2)	102.3 (10.1)	<0.001
SMI (kg/m^2^), median (IQR)	7.3 (6.3-7.9)	7.4 (7.0-8.1)	5.6 (5.3-6.3)	<0.001
Handgrip strength (kg), median (IQR)	27.9 (22.1-33.4)	29.9 (23.4-34.5)	19.3 (15.6-23.2)	<0.001
Gait speed, median (IQR)	1.1 (1.0-1.3)	1.2 (1.0-1.3)	0.9 (0.8-1.0)	<0.001
Preoperative BI score, median (IQR)	100 (100)	100 (100-100)	100 (100-100)	0.008
Mobility score, median (IQR)	15 (15-15)	15 (15-15)	15 (15-15)	1.000
Use of walking aids, n (%)	5 (3.1%)	2 (1.6%)	3 (8.8%)	0.030
VC (L), median (IQR)	3.3 (2.6-3.8)	3.3 (2.8-3.9)	2.6 (2.2-3.4)	0.069
FEV1 (L), median (IQR)	2.4 (1.9-2.7)	2.5 (2.1-2.9)	1.9 (1.6-2.5)	0.001
FEV1/FVC ratio (%), median (IQR)	74.7 (69.3-79.8)	75.2 (69.3-80.0)	71.5 (68.4-79.4)	0.586
Operating time (min), median (IQR)	350 (284-440)	350 (289-440)	333 (265-441)	0.484
Intraoperative blood loss (L), median (IQR)	2.2 (1.3-3.3)	2.2 (1.2-3.3)	2.1 (1.6-3.3)	0.778
Intraoperative fluid balance (L), median (IQR)	3.9 (2.9-5.4)	3.8 (2.9-5.4)	4.5 (2.9-5.7)	0.430
Postoperative complications, n (%)				
Renal failure requiring RRT	5 (3.1%)	4 (3.1%)	1 (2.9%)	0.950
Reoperation for bleeding	5 (3.1%)	4 (3.1%)	1 (2.9%)	0.950
Cardiac arrest	2 (1.2%)	0 (0%)	2 (5.9%)	0.006
Atrial fibrillation	48 (29.8%)	36 (28.3%)	12 (35.3%)	0.432
Prolonged mechanical ventilation	15 (9.3%)	8 (6.3%)	7 (20.6%)	0.011
Reintubation	6 (3.7%)	5 (3.9%)	1 (2.9%)	0.785
Tamponade	1 (0.6%)	0 (0%)	1 (2.9%)	0.053
Septicemia	3 (1.9%)	1 (0.8%)	2 (5.9%)	0.051
Gastrointestinal complication	1 (0.6%)	1 (0.8%)	0 (0%)	0.604
Composite complications	62 (38.5%)	45 (35.4%)	17 (50.0%)	0.121
Postoperative ventilation time (h), median (IQR)	12 (5-20)	9 (5-19)	16 (7-23)	0.079
Length of ICU stay (days), median (IQR)	4 (2-6)	4 (2-5)	5 (4-7)	0.001
Length of hospitalization (days), median (IQR)	12 (10-16)	12 (10-16)	14 (11-20)	0.081

BI: Barthel Index; BMI: body mass index; CABG: coronary artery bypass graft; eGFR: estimated glomerular filtration rate; FEV1: forced expiratory volume in one second; FVC: forced vital capacity; GNRI: geriatric nutritional risk index; ICU: intensive care unit; IQR: interquartile range; LVEF: left ventricular ejection fraction; NT-proBNP: N-Terminal pro-brain natriuretic peptide; NYHA: New York Heart Association; RRT: renal replacement therapy; SD: standard deviation; SMI: skeletal muscle mass index; VC: vital capacity.

**Figure 2.**
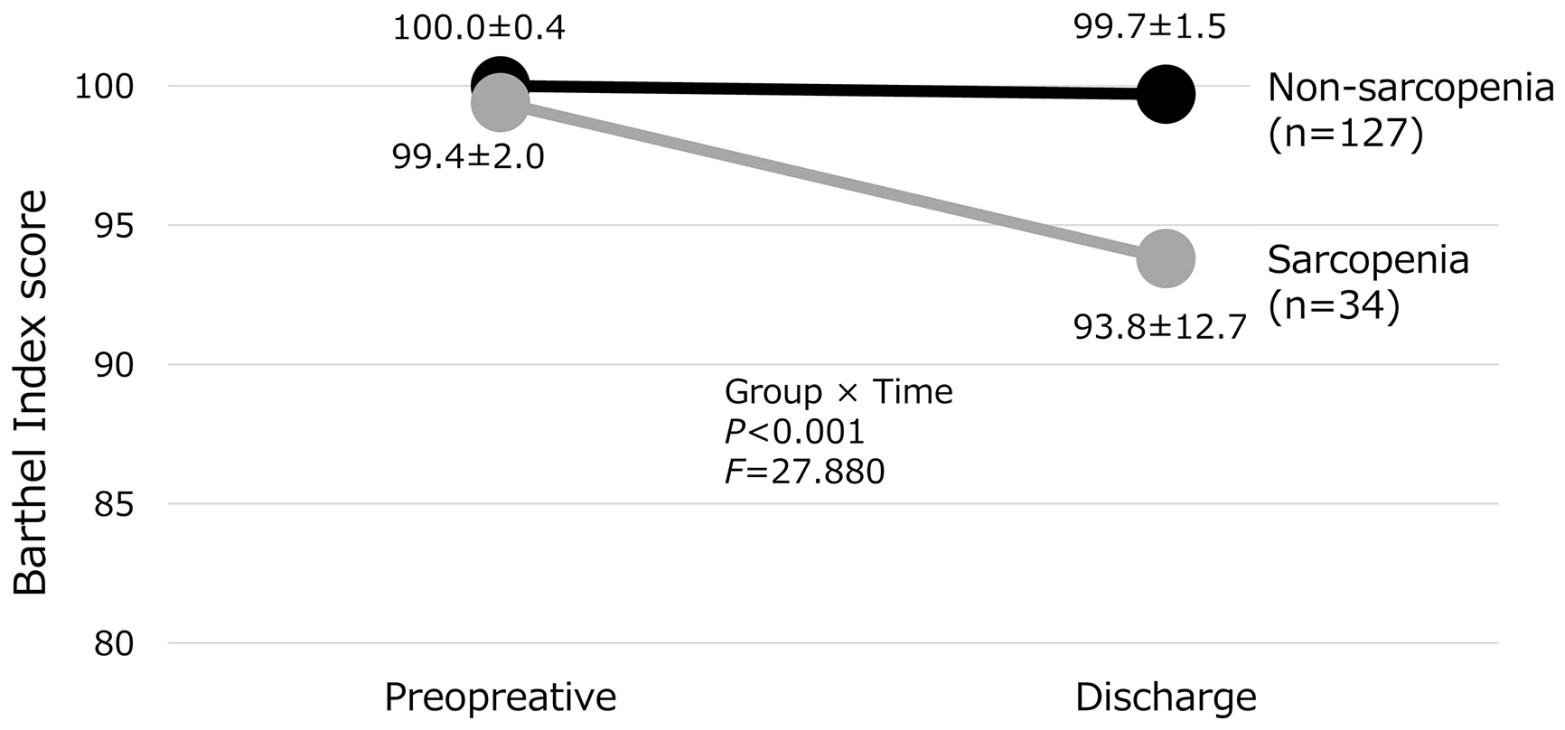
The changes in Barthel Index (BI) scores between the preoperative and discharge assessments in the non-sarcopenia and sarcopenia groups. In the two-way analysis of variance (ANOVA), a significant interaction was observed between the presence of sarcopenia and the time from preoperative to discharge.

The changes in BI score between the preoperative and discharge assessments in the non-sarcopenia and sarcopenia groups are shown in Figure 2. A significant interaction was observed between the presence of sarcopenia and the time (from preoperative to discharge) in the two-way ANOVA (p < 0.001).

**Figure 3.**
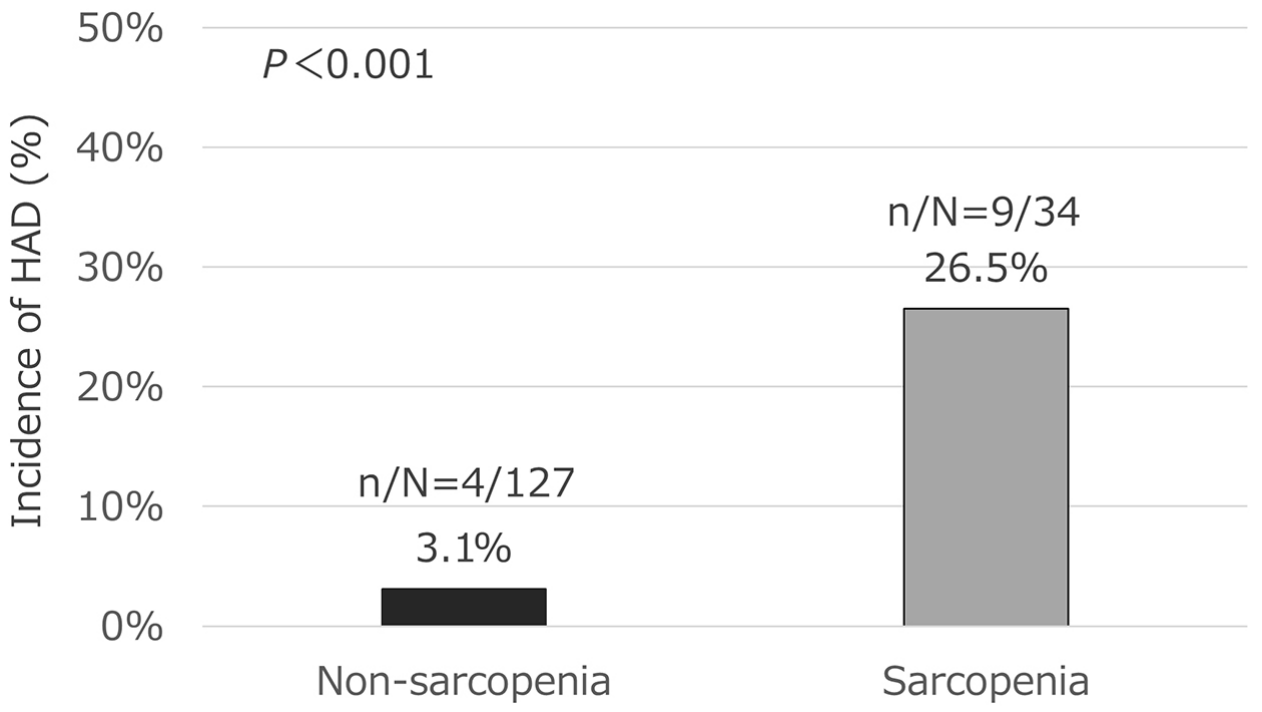
The incidence of hospitalization-associated disability (HAD) in non-sarcopenia and sarcopenia groups. The incidence of HAD was significantly higher in the sarcopenia group.

The incidence of HAD in the non-sarcopenia and sarcopenia groups is shown in Figure 3. The incidence of HAD (absolute number: n/N) was 3.1% (n/N = 4/127) and 26.5% (n/N = 9/34) in patients without and with sarcopenia, respectively. Significant differences in the incidence were observed (p < 0.001). Additionally, the incidence of HAD in patients with severe sarcopenia was 40.9% (n/N = 9/22). In contrast, the incidence of HAD in patients with (non-severe) sarcopenia was 0% (n/N = 0/12). Other variables that differed significantly between patients with and without incidence of HAD (p < 0.05) included age, heart failure, NYHA classification, SMI, handgrip strength, gait speed, preoperative BI score, use of walking aids, VC, FEV1, intraoperative fluid balance, complications (cardiac arrest, atrial fibrillation, prolonged mechanical ventilation, reintubation, tamponade, and composite complications), postoperative ventilation time, length of ICU stay, and length of hospitalization (Online Resource 2).

Data from the univariate and multivariate linear regression models are summarized in Table 2 and detailed results are shown in Online Resource 3. In the univariate linear regression analysis, sarcopenia was significantly associated with changes in the BI score (p < 0.001). Moreover, severe sarcopenia was significantly associated with changes in the BI score (p < 0.001). Other variables that were significantly associated with changes in the BI score in the univariate linear regression analyses included age, BMI, VC, FEV1, NYHA classification, preoperative BI score, use of walking aid, intraoperative fluid balance, postoperative complications (cardiac arrest, atrial fibrillation, prolonged mechanical ventilation, reintubation, and composite complications), postoperative ventilation time, and length of ICU stay (p < 0.05). After adjustment for age, sex, preoperative BI, NYHA class, VC, operative time, and composite postoperative complications, sarcopenia remained significantly associated with changes in the BI score (β = −0.185, p = 0.001). Moreover, severe sarcopenia demonstrated a stronger independent association with changes in the BI score (β = −0.268, p < 0.001).

**Table 2.** Univariate and Multivariate Linear Regression Analysis for Relationship between Change in Barthel Index Score and Sarcopenia or Severe Sarcopenia.

	Univariate			Multivariate			
	B (95% CI)	β	*P*	B (95% CI)	β	*P*	Adjusted R^2^
Sarcopenia	−5.352 (−7.354 to −3.350)	−0.386	<0.001	−2.569 (−4.046 to −1.093)	−0.185	0.001	0.612
Severe sarcopenia	−8.421 (−10.637 to −6.204)	−0.511	<0.001	−4.415 (−6.185 to −2.645)	−0.268	<0.001	0.640

Multivariate analyses were adjusted for age, sex, NYHA classification, preoperative BI, VC, operating time, and composite complications. BI: Barthel Index; CI: confidence interval; NYHA: New York Heart Association; VC: vital capacity.

## Discussion

The primary finding of this study was that preoperative sarcopenia was associated with postoperative functional decline in patients undergoing cardiac surgery, even after adjustment for relevant clinical confounders. In addition, severe sarcopenia tended to be associated with a higher risk of postoperative functional decline. This study provides additional evidence regarding the relationship among consensus-based sarcopenia, its severity, and postoperative functional outcomes in patients undergoing cardiac surgery.

The results of several previous studies support our findings ^(18), (27)^. Studies investigating the association between skeletal muscle mass and postoperative outcomes in patients undergoing cardiac surgery have demonstrated that a stepwise decline in muscle mass is significantly associated with increased mortality and in-hospital adverse events ^(18)^. Additionally, decreased skeletal muscle mass is reportedly associated with prolonged hospital stay ^(27)^. Although the methods for assessing sarcopenia and the evaluated outcomes differ among studies, our results are consistent, suggesting that sarcopenia severity may be linked to poorer postoperative outcomes.

A plausible mechanism underlying the increased risk of postoperative functional decline in patients with severe sarcopenia is the involvement of preoperative physical performance. Physical performance indicators, including gait speed, are associated with postoperative functional outcomes ^(6), (10), (11)^. In this study, patients classified into the severe sarcopenia group who showed a significantly higher risk of postoperative functional decline, had a significantly lower gait speed than those in the other two groups. In contrast, among patients with non-severe sarcopenia, all individuals maintained their BI scores at discharge and the majority (75%) exhibited gait speeds exceeding the reference threshold of 1.0 m/s. However, after adjustment for relevant clinical covariates, gait speed was no longer independently associated with changes in the BI score (Online Resource 4). These findings suggest that impaired physical performance may characterize patients with severe sarcopenia and may partially contribute to postoperative functional decline; however, its independent effect was not confirmed in the adjusted models.

A key strength of this study is the application of consensus-based diagnostic criteria and severity classification for sarcopenia, as proposed by the AWGS 2019, in patients undergoing cardiac surgery. Although previous studies have often focused solely on skeletal muscle mass using imaging-based measures, such as computed tomography-derived psoas area, this study assessed sarcopenia through multiple components, including muscle strength and physical performance (e.g., gait speed). This provides a more functionally relevant clinical perspective and incorporates measures that may be responsive to interventions ^(28)^. Outcomes were examined according to sarcopenia severity by distinguishing between non-severe and severe sarcopenia. Postoperative functional outcomes differed between these subgroups; HAD occurred more frequently in patients with severe sarcopenia, whereas no cases of HAD were observed in patients with non-severe sarcopenia. Although these findings should be interpreted with caution owing to the retrospective nature of the study and limited sample size, they suggest that sarcopenia severity may be relevant when assessing the risk of postoperative functional decline. Further studies are warranted to confirm these associations and to clarify their clinical implications.

This study had some limitations. First, as this was a single-center retrospective study, the generalizability of the findings should be interpreted with caution because of potential selection bias and missing data. Second, the ADL assessments were conducted over a short period; therefore, long-term outcomes require further investigation. Third, the relatively small sample size might have limited the robustness of the results. Although none of the patients in the non-severe sarcopenia group experienced HAD, the small sample size of this subgroup (n = 12) limited the statistical power and generalizability of this observation. Therefore, the absence of HAD in this group should be interpreted with caution, and future studies with larger cohorts are required to validate this finding. Fourth, selection bias may have influenced the results. In routine clinical practice, patients with severe preoperative disability are unlikely to be considered candidates for elective cardiac surgery. Consequently, individuals with markedly low baseline BI scores or severe functional impairment may have been underrepresented in this study. This clinical selection process may explain the relatively preserved preoperative BI scores observed even among patients classified as sarcopenic and may limit the generalizability of these findings to patients with more severe baseline disability. Finally, the impact of sarcopenia and HAD on prognosis could not be evaluated. During the follow-up period (mean: 571 days, standard deviation: 385 days), only one patient died after discharge. This patient had non-severe sarcopenia and was discharged without developing postoperative HAD. Consequently, evaluating the prognostic effects of sarcopenia and perioperative ADL in this study proved difficult. Future multicenter studies with larger cohorts or longer follow-up periods are warranted.

In conclusion, preoperative sarcopenia may be associated with postoperative functional decline in patients undergoing cardiac surgery. Severe sarcopenia appears to be associated with an increased risk of functional decline. These findings indicate that patients with severe sarcopenia require careful attention owing to their higher risk of postoperative functional decline.

## Article Information

### Acknowledgments

We thank the rehabilitation therapists at the Nippon Medical School Hospital for their contributions to data collection. We are grateful to Mr. Keisuke Aoki, Mr. Motomichi Masuyama, and Mr. Takayuki Yoshizawa for their insightful advice on the earlier drafts of this manuscript. We would like to thank Editage (www.editage.jp) for the English language editing.

### Author Contributions

All authors contributed to the conception and design of this study. Material preparation, data collection, and analyses were performed by Daisuke Ishiyama, Yoshiaki Kubota, and Masahiro Koen. Daisuke Ishiyama wrote the first draft of the manuscript, and all authors commented on the previous versions of the manuscript and have also read and approved the final version of the manuscript.

### Conflicts of Interest

None

### IRB Approval Code and Name of the Institution

The study was conducted in accordance with the principles of the Declaration of Helsinki. The study protocol was reviewed and approved by the Institutional Committee on Human Research at the study facility (approval no. B-2020-325). Informed consent for this study was obtained using the opt-out method of poster announcements at Nippon Medical School Hospital.

### Data Transparency

The data are not publicly available due to ethical restrictions but are available from the corresponding author upon reasonable request.

## Supplement

Supplementary Material
