# Supplementary material for: Impact of Sarcopenia and Its Severity on Postoperative Functional Decline in Patients Undergoing Cardiac Surgery
Source: JMA J. 2026 May 8;9(4):909–16. doi: 10.31662/jmaj.2026-0041 (PMC13453903; doi:10.31662/jmaj.2026-0041)
Supplement: Supplementary file 1 [file 2433-3298-9-4-0909-s001.pdf]

Supplementary material

Article title

Impact of Sarcopenia and Its Severity on Postoperative Functional Decline in Patients Undergoing Cardiac Surgery

Journal name

JMA journal

Daisuke Ishiyama[1)], Yoshiaki Kubota[2)], Masahiro Koen[2)], Aya Moroi[1)],

Momoe Ono[1)], Hayato Watanabe[1)], Kaito Sakai[1)], Yukihiro Watanabe[2)],

Hitomi Ueda[3)], Kuniya Asai[2)], Yosuke Ishii[3)], and Yoichiro Aoyagi[1)]

[1)] Department of Rehabilitation Medicine, Nippon Medical School Hospital, Tokyo, Japan

[2)] Department of Cardiovascular Medicine, Nippon Medical School, Tokyo, Japan

[3)] Department of Cardiovascular Surgery, Nippon Medical School, Tokyo, Japan

Corresponding author: Daisuke Ishiyama, d-ishiyama@nms.ac.jp

Online Resource 1. Patient Characteristics According to Sarcopenia Severity

	Overall (n=161)	Non-sarcopenia (n=127)	Sarcopenia (n=12)	Severe sarcopenia (n=22)	*P*	Post hoc tests
Age (year), median (IQR)	71 (64-75)	70 (61-74)	72 (66-75)	75 (71-80)	0.001	1-3
Sex, male, n (%)	122 (75.8%)	99 (79.2%)	7 (58.3%)	14 (63.6%)	0.094	
Underlying disease, n (%)					0.419	
Ischemic heart disease	53 (32.9%)	45 (35.4%)	3 (25.0%)	5 (22.7%)		
Valvular disease	108 (67.1%)	82 (64.6%)	9 (75.0%)	17 (77.3%)		
Cardiac procedures, n (%)					0.704	
Isolated CABG	51 (31.7%)	43 (33.9%)	3 (25.0%)	5 (22.7%)		
Isolated heart valve surgery	68 (42.2%)	51 (40.2%)	5 (41.7%)	12 (54.5%)		
Combined	42 (26.1%)	33 (26.0%)	4 (33.3%)	5 (22.7%)		
BMI (kg/m^2), median (IQR)	23.7 (21.6-25.6)	24.3 (22.1-26.5)	21.7 (18.9-23.1)	20.7 (18.4-23.0)	<0.001	1-2, 1-3
Comorbidity, n (%)						
Hypertension	91 (56.5%)	73 (57.5%)	5 (41.7%)	13 (59.1%)	0.553	
Dyslipidemia	78 (48.4%)	64 (50.4%)	2 (16.7%)	12 (54.5%)	0.068	
Diabetes	45 (28.0%)	36 (28.3%)	4 (33.3%)	8 (36.4%)	0.215	
Stroke	18 (11.2%)	11 (8.7%)	1 (8.3%)	6 (27.3%)	0.036	
Heart failure	37 (23.0%)	21 (16.5%)	7 (58.3%)	9 (40.9%)	<0.001	
NYHA classification, n (%)					0.005	
I	40 (24.8%)	37 (29.1%)	3 (25.0%)	0 (0%)		
II	118 (73.3%)	89 (70.1%)	9 (75.0%)	20 (90.9%)		
III	3 (1.9%)	1 (0.8%)	0 (0%)	2 (9.1%)		
LVEF [%), median (IQR)	64 (53-69)	64 (54-69)	63 (50-76)	49 (36-64)	0.623	
NT-proBNP [pg/mL), median (IQR)	486 (159-1260)	447 (122-1136)	577 (251-1862)	1044 (304-2477)	<0.001	1-3
eGFR (mL/min/1.73m^2), median (IQR)	60 (43-70)	62 (48-70)	41 (36-82)	54 (39-60)	0.081	
Serum albumin (g/dL), median (IQR)	4.3 (4.0-4.5)	4.3 (4.0-4.6)	4.2 (4.0-4.5)	4.3 (3.9-4.5)	0.435	

GNRI, mean (SD)	109.4 (9.9)	110.0 (9.2)	105.5 (9.0)	102.7 (10.8)	<0.001	1-2, 1-3
SMI (kg/m^2), median (IQR)	7.3 (6.3-7.9)	7.4 (7.0-8.1)	5.6 (5.2-6.5)	5.7 (5.3-6.3)	<0.001	1-2, 1-3
Handgrip strength (kg), median (IQR)	27.9 (22.1-33.4)	29.9 (23.4-34.5)	23.1 (17.2-27.3)	17.2 (15.4-22.6)	<0.001	1-2, 1-3
Gait speed (m/s), median (IQR)	1.1 (1.0-1.3)	1.2 (1.0-1.3)	1.2 (1.0-1.4)	0.9 (0.7-0.9)	<0.001	1-3, 2-3
Preoperative BI score, median (IQR)	100 (100)	100 (100-100)	100 (100-100)	100 (100-100)	0.001	1-3, 2-3
Mobility score, median (IQR)	15 (15-15)	15 (15-15)	15 (15-15)	15 (15-15)	1.000	
Use of walking aids, n (%)	5 (3.1%)	2 (1.6%)	0 (0%)	3 (13.6%)	0.009	
VC (L), median (IQR)	3.3 (2.6-3.8)	3.3 (2.8-3.9)	3.3 (2.2-3.6)	2.4 (2.1-2.9)	<0.001	1-3
FEV1 (L), median (IQR)	2.4 (1.9-2.7)	2.5 (2.1-2.9)	2.5 (1.7-2.5)	1.7 (1.5-2.2)	<0.001	1-3
FEV1/FVC ratio (%), median (IQR)	74.7 (69.3-79.8)	75.2 (69.3-80.0)	70.4 (67.6-78.2)	73.4 (69.3-80.6)	0.623	
Operating time (min), median (IQR)	350 (284-440)	350 (289-440)	339 (283-420)	321 (250-443)	0.761	
Intraoperative blood loss (L), median (IQR)	2.2 (1.3-3.3)	2.2 (1.2-3.3)	2.2 (1.9-3.1)	1.9 (1.2-3.6)	0.929	
Intraoperative fluid balance (L), median (IQR)	3.9 (2.9-5.4)	3.8 (2.9-5.4)	3.6 (1.7-5.7)	4.6 (3.2-5.7)	0.333	
Postoperative complications, n (%)						
Renal failure requiring RRT	5 (3.1%)	4 (3.1%)	1 (8.3%)	0 (0%)	0.407	
Reoperation for bleeding	5 (3.1%)	4 (3.1%)	0 (0%)	1 (4.5%)	0.765	
Cardiac arrest	2 (1.2%)	0 (0%)	0 (0%)	2 (9.1%)	0.002	
Atrial fibrillation	48 (29.8%)	36 (28.3%)	3 (25.0%)	9 (40.9%)	0.459	
Prolonged mechanical ventilation	15 (9.3%)	8 (6.3%)	2 (16.7%)	5 (22.7%)	0.033	
Reintubation	6 (3.7%)	5 (3.9%)	0 (0%	1 (4.5%)	0.771	
Tamponade	1 (0.6%)	0 (0%)	0 (0%)	1 (4.5%)	0.042	
Septicemia	3 (1.9%)	1 (0.8%)	2 (16.7%)	0 (0%)	<0.001	
Gastrointestinal complication	1 (0.6%)	1 (0.8%)	0 (0%)	0 (0%)	0.874	
Composite complications	62 (38.5%)	45 (35.4%)	4 (33.3%)	13 (59.1%)	0.101	
Postoperative ventilation time (h), median (IQR)	12 (5-20)	9 (5-19)	14 (5-19)	17 (8-56)	0.097	
Length of ICU stay (days), median (IQR)	4 (2-6)	4 (2-5)	5 (3-7)	5 (4-7)	0.003	1-3
Length of hospitalization (days), median (IQR)	12 (10-16)	12 (10-16)	15 (9-19)	16 (12-21)	0.146	

SD: standard deviation; IQR: interquartile range; CABG: coronary artery bypass graft; BMI: body mass index; NYHA: New York Heart Association; LVEF: left ventricular ejection fraction; NT-proBNP: N-Terminal pro brain natriuretic peptide; eGFR: estimated glomerular filtration rate; GNRI: geriatric nutritional risk index; SMI: skeletal muscle mass index; BI: Barthel Index; VC: vital capacity; FEV1: forced expiratory volume in one second; FVC: forced vital capacity; RRT: renal replacement therapy; ICU: intensive care unit.

Numbers indicate group combinations for which significant differences were observed in post hoc tests.: 1, non-sarcopenia; 2, sarcopenia; 3, severe sarcopenia.

Online Resource 2. Patient Characteristics According to Hospitalization-Associated Disability.

	Non-HAD (n=148)	HAD (n=13)	*P*
Age (year), median (IQR)	70 (62-74)	75 (73-78)	0.001
Sex, male, n (%)	114 (77.0%)	8 (61.5%)	0.211
Underlying disease, n (%)			0.431
Ischemic heart disease	50 (33.8%)	3 (23.1%)	
Valvular disease	98 (66.2%)	10 (76.1%)	
Cardiac procedures, n (%)			0.055
Isolated CABG	49 (33.1%)	2 (15.4%)	
Isolated heart valve surgery	64 (43.2%)	4 (30.8%)	
Combined	35 (23.6%)	7 (53.8%)	
BMI (kg/m^2), median (IQR)	23.8 (21.6-25.7)	21.9 (19.8-24.3)	0.079
Comorbidity, n (%)			
Hypertension	81 (54.7%)	10 (76.9%)	0.122
Dyslipidemia	70 (47.3%)	8 (61.5%)	0.325
Diabetes	40 (27.0%)	5 (38.5%)	0.378
Stroke	16 (10.8%)	2 (15.4%)	0.616
Heart failure	31 (20.9%)	6 (46.2%)	0.038
NYHA classification, n (%)			<0.001
I	39 (26.4%)	1 (7.7%)	
II	73 (73.0%)	10 (76.9%)	
III	1 (0.7%)	2 (15.4%)	
LVEF (%), median (IQR)	64 (53-69)	63 (52-72)	0.955
NT-proBNP (pg/mL), median (IQR)	450 (153-1236)	1201 (420-2689)	0.013
eGFR (mL/min/1.73m^2), median (IQR)	61 (44-71)	49 (33-54)	0.002

Serum albumin (g/dL), median (IQR)	4.3 (4.0-4.5)	4.4 (3.7-4.6)	0.674
GNRI, mean (SD)	108.8 (9.7)	103.9 (11.0)	0.089
SMI (kg/m^2), median (IQR)	7.3 (6.5-7.9)	5.9 (5.2-6.5)	0.003
Handgrip strength (kg), median (IQR)	28.4 (22.6-33.6)	17.6 (15.5-23.1)	<0.001
Gait speed, (m/s), median (IQR)	1.2 (1.0-1.3)	0.9 (0.7-1.0)	<0.001
Sarcopenia severity, n (%)			<0.001
Non-sarcopenia	123 (83.1%)	4 (30.8%)	
Sarcopenia	12 (8.1%)	0 (0%)	
Severe Sarcopenia	12 (8.8%)	9 (69.2%)	
Preoperative BI score, median (IQR)	100 (100-100)	100 (98-100)	<0.001
Mobility score, median (IQR)	15 (15-15)	15 (15-15)	1.000
Use of walking aids, n (%)	1 (0.7%)	4 (30.8%)	<0.001
VC (L), median (IQR)	3.3 (2.7-3.8)	2.1 (2.1-2.8)	<0.001
FEV1 (L), median (IQR)	2.4 (2.0-2.9)	1.6 (1.6-2.1)	<0.001
FEV1/FVC ratio (%), median (IQR)	74.9 (69.1-79.9)	73.6 (70.5-79.5)	0.894
Operating time (min), median (IQR)	348 (285-430)	395 (277-561)	0.178
Intraoperative blood loss (L), median (IQR)	2.1 (1.3-3.2)	3.3 (1.5-5.3)	0.075
Intraoperative fluid balance (L), median (IQR)	3.8 (2.8-5.1)	5.7 (5.2-6.7)	0.003
Postoperative complications, n (%)			
Renal failure requiring RRT	4 (2.7%)	1 (7.7%)	0.320
Reoperation for bleeding	4 (2.7%)	1 (7.7%)	0.320
Cardiac arrest	0 (0%)	2 (15.4%)	<0.001
Atrial fibrillation	39 (26.4%)	9 (69.2%)	0.001
Prolonged mechanical ventilation	9 (6.1%)	6 (46.2%)	<0.001
Reintubation	2 (1.4%)	4 (30.8%)	<0.001
Tamponade	0 (0%)	1 (7.7%)	0.001
Septicemia	3 (2.0%)	0 (0%)	0.604

Gastrointestinal complication	1 (0.7%)	0 (0%)	0.766
Composite complications	50 (33.8%)	12 (92.3%)	<0.001
Postoperative ventilation time (h), median (IQR)	9 (5-19)	63 (12-95)	0.002
Length of ICU stay (days), median (IQR)	4 (2-5)	7 (4-16)	<0.001
Length of hospitalization (days), median (IQR)	12 (10-16)	21 (17-39)	<0.001

HAD: hospitalization-associated disability; SD: standard deviation; IQR: interquartile range; CABG: coronary artery bypass graft; BMI: body mass index; NYHA: New York Heart Association; LVEF: left ventricular ejection fraction; NT-proBNP: N-Terminal pro brain natriuretic peptide; eGFR: estimated glomerular filtration rate; GNRI: geriatric nutritional risk index: SMI, skeletal muscle mass index; BI: Barthel Index; VC: vital capacity; FEV1: forced expiratory volume in one second; FVC: forced vital capacity; RRT: renal replacement therapy; ICU: intensive care unit.

Online Resource 3. Detailed Results of Linear Regression Analysis

	Univariate			Multivariate (Sarcopenia model)			Multivariate (Severe sarcopenia model)		
	B (95%CI)	β	*P*	B (95%CI)	β	*P*	B (95%CI)	β	*P*
Sarcopenia	-5.352 (-7.354 to -3.350)	-0.386	<0.001	-2.569 (-4.046 to -1.093)	-0.185	0.001			
Severe sarcopenia	-8.421 (-10.637 to -6.204)	-0.511	<0.001				-4.415 (-6.185 to -2.645)	-0.268	<0.001
Age (year)	-0.089 (-0.165 to -0.014)	-0.182	0.021	-0.016 (-0.074 to 0.042)	-0.032	0.595	-0.011 (-0.067 to 0.045)	-0.023	0.687
Sex (male)	1.411 (-0.644 to 3.467)	0.107	0.177	-1.837 (-3.524 to -0.150)	-0.139	0.033	-1.442 (-3.075 to 0.191)	-0.109	0.083
Underlying disease (valvular)	-0.912 (-2.792 to 0.968)	-0.076	0.339						
Cardiac procedures									
Isolated CABG	1.000 (reference)								
Isolated heart valve surgery	-0.490 (-2.563 to -1.582)	-0.043	0.641						
Combined	-1.814 (-4.145 to 0.517)	-0.141	0.126						
BMI (kg/m^2)	0.243 (0.004 to 0.482)	0.157	0.046						
Hypertension	-1.533 (-3.304 to 0.238)	-0.134	0.089						
Dyslipidemia	-0.458 (-2.229 to 1.313)	-0.040	0.610						
Diabetes	-0.417 (-2.389 to 1.556)	-0.033	0.677						
Stroke	-1.276 (-4.080 to 1.528)	-0.071	0.370						
Heart failure	-0.858 (-2.959 to 1.243)	-0.064	0.421						
NYHA classifications (II-III)									
I	1.000 (reference)			1.000 (reference)			1.000 (reference)		
II	-0.894 (-2.575 to 0.787)	-0.070	0.295	-0.131 (-1.470 to 1.208)	-0.010	0.847	-0.086 (-1.211 to 1.383)	-0.007	0.896
III	-24.750 (-30.249 to -19.251)	-0.592	<0.001	-16.195 (-20.990 to -11.399)	-0.387	<0.001	-15.212 (-19.865 to -10.559)	-0.364	<0.001
LVEF (%)	0.008 (0.059 to 0.074)	0.018	0.821						
NT-proBNP (per 100 pg/ml)	-0.003 (-0.008 to 0.003)	-0.074	0.353						
eGFR (mL/min/1.73m^2)	0.037 (-0.004 to 0.077)	0.141	0.074						
Serum albumin (g/dl)	-0.164 (-2.239 to 1.912)	-0.012	0.877						
GNRI	0.059 (0.030 to 0.149)	0.103	0.193						

Preoperative BI score	3.453 (2.785 to 4.120)	0.629	<0.001	2.143 (1.532 to 2.755)	0.391	<0.001	2.018 (1.424 to 2.612)	0.368	<0.001
Use of walking aids	-17.167 (-21.508 to -12.826)	-0.527	<0.001						
VC (L)	1.913 (0.874 to 2.951)	0.277	<0.001	1.200 (0.248 to 2.152)	0.174	0.014	0.929 (-0.002 to 1.860)	0.134	0.050
FEV1 (L)	2.073 (0.735 to 3.411)	0.236	0.003						
FEV1/FVC ratio (%)	-0.054 (0.141 to 0.033)	-0.097	0.222						
Operating time (min)	-0.007 (-0.015 to 0.002)	-0.127	0.109	-0.009 (-0.015 to -0.004)	-0.177	0.001	-0.009 (-0.014 to -0.003)	-0.164	0.001
Intraoperative blood loss (L)	-0.469 (-1.082 to 0.145)	-0.119	0.134						
Intraoperative fluid balance (L)	-0.552 (-0.982 to -0.123)	-0.197	0.012						
Postoperative complications									
Renal failure requiring RRT	-0.654 (-5.759 to 4.451)	-0.020	0.801						
Reoperation for bleeding	-0.654 (-5.759 to 4.451)	-0.020	0.801						
Cardiac arrest	-16.336 (-23.913 to -8.760)	-0.320	<0.001						
Atrial fibrillation	-2.357 (-4.258 to -0.456)	-0.191	0.015						
Prolonged mechanical ventilation	-5.845 (-8.751 to -2.938)	-0.300	<0.001						
Reintubation	-7.237 (-11.774 to -2.700)	-0.242	0.002						
Tamponade	-8.688 (-19.880 to 2.505)	-0.121	0.127						
Septicemia	1.392 (-5.154 to 7.939)	0.033	0.675						
Gastro-intestinal complication	1.375 (-9.897 to 12.647)	0.019	0.810						
Composite complications	-3.286 (-5.032 to -1.540)	-0.283	<0.001	-1.333 (-0.015 to -0.004)	-0.115	0.031	-1.259 (-2.425 to -0.094)	-0.108	0.034
Postoperative ventilation time (h)	-0.030 (-0.047 to -0.013)	-0.269	0.001						
Length of ICU stay (days)	-0.399 (-0.598 to 0.201)	-0.301	<0.001						
Adjusted R^2				0.612			0.640		

CI: confidence interval; CABG: coronary artery bypass graft; BMI: body mass index; NYHA: New York Heart Association; LVEF: left ventricular ejection fraction; NT-proBNP: N-Terminal pro brain natriuretic peptide: eGFR, estimated glomerular filtration rate: GNRI: geriatric nutritional risk index; BI: Barthel Index; VC: vital capacity; FEV1: forced expiratory volume in one second; FVC: forced vital capacity; RRT: renal replacement therapy; ICU: intensive care unit

Online Resource 4. Linear Regression Analysis for Relationship between Change in BI Score and Components of Sarcopenia.

	Univariate			Multivariate			
	B (95%CI)	β	*P*	B (95%CI)	β	*P*	Adjusted R^2
Low SMI	-3.990 (-5.882 to -2.157)	-0.323	<0.001	-1.938 (-3.309 to -0.567)	-0.157	0.006	0.602
Low handgrip strength	-3.101 (-4.850 to -1.352)	-0.268	0.001	-0.731 (-2.018 to 0.556)	-0.063	0.264	0.585
Low gait speed	-4.022 (-5.879 to -2.165)	-0.321	<0.001	-1.109 (-2.565 to 0.347)	-0.089	0.134	0.588

Multivariate analyses were adjusted for age, sex, NYHA classification, preoperative BI, VC, operating time, composite complications.

CI: confidence interval
